# Phylogenetic Analysis of Microbial Communities in Different Regions of the Gastrointestinal Tract in *Panaque nigrolineatus*, a Wood-Eating Fish

**DOI:** 10.1371/journal.pone.0048018

**Published:** 2012-10-25

**Authors:** Ryan McDonald, Harold J. Schreier, Joy E. M. Watts

**Affiliations:** 1 Department of Biological Sciences, Towson University, Towson, Maryland, United States of America; 2 Department of Marine Biotechnology and Department of Biological Sciences, University of Maryland Baltimore County, Baltimore, Maryland, United States of America; 3 Department of Biological Sciences, Portsmouth University, Portsmouth, United Kingdom; Institute for Genome Sciences, University of Maryland School of Medicine, United States of America

## Abstract

The Neotropical detritivorous catfish *Panaque nigrolineatus* imbibes large quantities of wood as part of its diet. Due to the interest in cellulose, hemi-cellulose and lignin degradation pathways, this organism provides an interesting model system for the detection of novel microbial catabolism. In this study, we characterize the microbial community present in different regions of the alimentary tract of *P. nigrolineatus* fed a mixed diet of date palm and palm wood in laboratory aquaria. Analysis was performed on 16S rRNA gene clone libraries derived from anterior and posterior regions of the alimentary tract and the auxiliary lobe (AL), an uncharacterized organ that is vascularly attached to the midgut. Sequence analysis and phylogenetic reconstruction revealed distinct microbial communities in each tissue region. The foregut community shared many phylotypes in common with aquarium tank water and included *Legionella* and *Hyphomicrobium* spp. As the analysis moved further into the gastrointestinal tract, phylotypes with high levels of 16S rRNA sequence similarity to nitrogen-fixing *Rhizobium* and *Agrobacterium* spp. and *Clostridium xylanovorans* and *Clostridium saccharolyticum,* dominated midgut and AL communities. However, the hindgut was dominated almost exclusively by phylotypes with the highest 16S rRNA sequence similarity to the *Cytophaga*-*Flavobacterium*-*Bacteroides* phylum. Species richness was highest in the foregut (Chao_1_ = 26.72), decreased distally through the midgut (Chao_1_ = 25.38) and hindgut (Chao_1_ = 20.60), with the lowest diversity detected in the AL (Chao_1_ = 18.04), indicating the presence of a specialized microbial community. Using 16S rRNA gene phylogeny, we report that the *P. nigrolineatus* gastrointestinal tract possesses a microbial community closely related to microorganisms capable of cellulose degradation and nitrogen fixation. Further studies are underway to determine the role of this resident microbial community in *Panaque nigrolineatus*.

## Introduction

*Loricariidae* catfish are a diverse group, consisting of over 600 species found predominantly in freshwater ecosystems of the Neotropics [1]. The *Loricariidae* inhabit a wide range of trophic levels and are generally believed to be omnivorous [2]. The xylivorous *Loricariidae* have unique musculature around the suckermouth and robust, fully mineralized, spoon-shaped teeth [2], [3], [4]; both adaptations are believed to allow the fish to adhere to and ingest submerged woody materials. Stable isotope evidence supports wood ingestion throughout the lifetime of the fish [5], [6], which may also provide a selective advantage during the Amazonian dry season when river nutrient input is limited [7].

Analysis of the gut content of several species of *Panaque* indicates that wood constitutes the majority (up to 75%) of the digesta from fish in the field [8], [9], [10]. However, recent studies examining gut transit time, digestive enzyme activity levels, and concentration of fermentative end-products have determined that *Panaque* are detritivores and do not obtain energy from the digestion of wood [9], [10]. Although the fish cannot digest wood directly, they imbibe microbes associated with the wood and microbial by-products produced during wood breakdown within the GI tract [6]. However, the inability to detect a resident microbial community using microscopy [9] raised interesting questions about this ecological niche and its colonization. *Panaque* contain a long GI tract, which is as much as ten times body length [11], providing many different microenvironments. Highly enriched in cellulose and other wood components, the *P. nigrolineatus* GI tract provides a novel environment with the potential to yield new cellulose degrading microorganisms and pathways.

Due to its ubiquity, microbial cellulose utilization is one of the largest energy flow pathways in the biosphere [12]. Cellulose degradation is a widely distributed activity within bacterial genera [13], and can occur either aerobically or anaerobically [14]. Although cellulose degradation activities have been detected in a wide range of microbes, it requires specialized enzymes due to the presence of *β*-1,4 glycosidic linkages that join the repeating glucose monomers [15]. Other biopolymers present in wood (e.g. lignin) increase cellulose recalcitrance to microbial attack [16].

The nitrogen-limiting nature of wood also poses a physiological challenge to xylivorous organisms. The nitrogen content of mature structural wood is significantly less (0.5–1.5% as litter) [17] than that of primary consumers (5.6–12.6% dry weight) [18]. Therefore, xylivorous organisms must supplement their diet with additional nitrogen sources or selectively eliminate carbonaceous compounds from their body. For all well characterized xylophagic systems, including marine wood-boring bivalves and lower termites, the former scenario holds true, with each possessing at least one endosymbiotic bacterial species capable of nitrogen fixation [19], [20]. These symbionts reduce atmospheric molecular nitrogen to ammonia that can be assimilated by the host. The nature of these symbioses are highly variable and range from very narrow communities housed in specialized tissues (shipworms and *Tetraponera* ants) [21], [22], [23] to complex mixed microbial communities located throughout the entirety of the GI tract (termites) [24]. Previous studies examining the nitrogen balance in *P. nigrolineatus* revealed higher levels of nitrogen in the waste than the ingested wood [9] and an increase in microbially fixed nitrogen within fish tissue using stable isotopes [6], underscoring the interest in any resident diazatrophic community.

In this study, we examined 16S rRNA gene clone libraries created from the microbial communities associated with the foregut, midgut, hindgut, and auxiliary lobe (AL) of *P. nigrolineatus*. Our results reveal the presence of diverse and different communities in these GI tract regions, providing insight into the diversity of microbial community and identifying members having the potential to degrade cellulose and fix molecular nitrogen.

## Materials and Methods

### Study Organisms and Acclimation

*Panaque nigrolineatus* (L-190) were imported from the Peruvian Amazon jungle basin from the fish wholesaler Aquascapesonline (Belleville, NJ) without antibiotics. Fish were acclimated in individual, filtered and aerated tanks, kept at a temperature of 29±1°C. Fish were fed a mixed diet of hearts of palm (*Euterpe precatoria*), algae pellets (Hikari Tropical Sinking Algae Wafers®, Hayward, CA), and date palm wood (*Phoenix dactylifera*) during an acclimation period of three weeks. Fish were randomly assigned to tanks and fish smaller than 40 mm (standard length) were excluded from the analysis. For the duration of the experiment, fish were fed a mixed diet in which palm hearts and algae were provided every second day while wood was constantly available. To eliminate contamination and to facilitate accessibility to the fish via water saturation, wood was thoroughly soaked in water and autoclaved three times prior to placement in the tank. Fish were reared under dark/low light conditions to inhibit algal growth, although tanks were open to the environment under non-sterile conditions. Fish were maintained under these conditions for four weeks prior to termination.

### Ethics Statement

Fish were maintained and sacrificed under strict accordance with the recommendations of the IACUC protocol to minimize suffering, approved by the Committee on the Ethics of Animal Experiments of the University of Maryland (071509JW-01).

### Tissue Sampling

At the conclusion of the feeding period, three fish were sacrificed by anesthetic overdose in 50 mg L^−1^ 3-aminobenzoic acid ethyl ester (MS-222), (Sigma Chemical Co., St. Louis, MO). Animals were dissected immediately; as soon as the ventral body plate was removed, ice cold sterile phosphate buffered saline (PBS) was added to the abdominal cavity and 1.0 cm sections of the foregut, midgut, and hindguts as well as the whole AL were collected. Any digesta and associated bacteria present in the lumen of the sampled tissue was included in subsequent analyses. From the tanks that had housed the fish 160 ml of water and sediment were collected. The tank water/sediment was centrifuged (10,000 g for 15 min.) and the supernatant was discarded. The pellet and tissue sections were stored at −80°C prior to DNA extraction.

### Tissue Digestion and Microbial DNA Extraction

Tissue samples were digested using the Roche High Pure Template Preparation Kit (Indianapolis, IN). The protocol was modified to include steps from both mammalian and bacterial DNA extraction. Briefly, tissue (including digesta) was incubated in 200 µl tissue lysis buffer and 40 µl proteinase K solution for one hr at 55°C. To this solution, 5 µl lysozyme (10 mg/ml) was added and incubated for 15 min. at 37°C. Finally, 200 µl binding buffer and 40 µl proteinase K solution was added followed by a 10 min. incubation at 70°C.

### Cloning and Bacterial Transformation

Microbial community 16S rRNA genes were amplified using universal bacterial primers 27F (5′-AGAGTTTGATCMTGGCTCAG-3′) [25] and 1392R (5′-ACGGGCGGTGTGTAC-3′) [26]. Amplification was performed using a BioRad MJ Mini thermal cycler with GoTaq Green Master Mix (Promega, Madison, WI). PCR program parameters were: initial denaturation step of 3 min. at 94°C followed by 30 cycles of denaturation for 1 min at 94°C, annealing for 1.5 min at 58°C, elongation for 1.5 min at 72°C, followed by a final elongation for 10 min at 72°C. The size and yield of the PCR products were verified by gel electrophoresis. Amplified 16S rRNA genes were ligated into pCR 2.1 vector (Invitrogen, Carlsbad, CA) according to the manufacturer’s instructions and ligation products were transformed into One Shot Top 10 chemically competent cells (Invitrogen, Carlsbad, CA).

### Sequencing and Phylogenetic Analysis

Clones from each fish tissue region were pooled to yield between 100–110 clones per library. Plasmid preparation and sequencing was performed by Functional Biosciences, Inc. (Madison, WI). Sequences were trimmed automatically using a Phred quality score of 20 as the threshold, followed by manual editing. Contigs were assembled using the CAP3 contig assembly program (Huang and Madan, 1999) and checked for chimeric sequences using Greengenes [27]. Full contigs were used as search queries on Basic Local Alignment Search Tool (BLAST) [28]. The search was optimized for somewhat similar sequences (BLASTn) and environmental and metagenomic sequences were eliminated from the search. Each unknown operational taxonomic unit (OTU) was binned according the percent similarity to previously uploaded sequences. Sequences were aligned pairwise to *E. coli* automatically in PHYDIT (Chun, 1995) followed by manual adjustment. Aligned sequences were imported into MacClade version 4.0 (Maddison and Maddison, 2000) to generate the PAUP block necessary for phylogenetic analysis. Phylogenies were reconstructed by Bayesian inference using the MrBayes program [29]. Each clone library was analyzed independently with the number of generations and burn-in value determined by the number of generations to reach stationarity. Sequences were deposited in GenBank with accession numbers JF681377–JF681782.

### Diversity Indices and Rarefaction Analysis

To examine changes in community composition across tissue regions, OTUs were binned to genus according to their closest match in GenBank and analyzed by different diversity indices. Simpson’s Diversity Index (D) [30] and Choa_1_ estimators [31] were applied, allowing non-parametric estimates of species richness [32], [33]. Clone library community coverage was determined using values calculated from the Chao_1_ estimates by dividing the observed genera by the predicted richness. As an additional means of assessing biodiversity, rarefaction analyses were also performed allowing comparisons of species richness across communities with different sample sizes by plotting species richness as a function of the number of sequences.

## Results

### 16S rRNA Gene Library Distribution and Diversity

**Figure 1:**
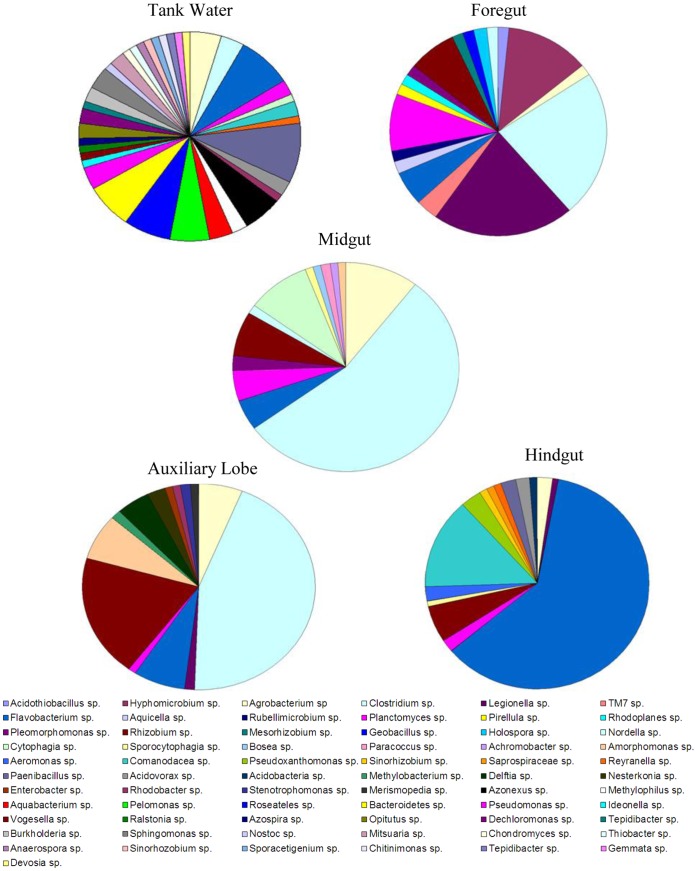
Abundance of bacterial genera from the various GI tract regions of *P. nigrolineatus*. Relative abundance of bacterial genera identified by 16S rRNA gene cloning recovered from the various GI tract regions of *P. nigrolineatus*. OTUs were binned to genus according to their closest match in GenBank.

Each 16S rRNA gene clone library was sequenced and analyzed for similarity using BLASTn [28] (Supplementary Tables 1–5). From this analysis OTUs were binned into taxonomic groupings allowing comparison of the different tissues (Fig. 1). Comparing OTU distributions for the different tissue types (Fig. 1), it can be observed that water had the highest diversity of OTUs and that the composition of the microbial community changed according to tissue type. Microbial community diversity decreased as samples were obtained from distal portions of the GI tract, with the midgut, hindgut and AL all displaying very different and specialized microbial communities (Fig. 1). Midgut- and AL-derived clone libraries were both dominated by sequences most closely related to *Clostridia* spp. and the *Rhizobium/Agrobacterium* group of *Alphaproteobacteria*. The 16S rRNA gene library derived from the hindgut appeared to be markedly different in composition to the midgut and AL, and was dominated by *Flavobacterium* sp. Some bacterial groups were present throughout the GI tract in low levels such as *Planctomycetes* sp., *Rhizobium* sp., *Agrobacterium* sp., and other members of the *Alphaproteobacteria.*

**Figure 2:**
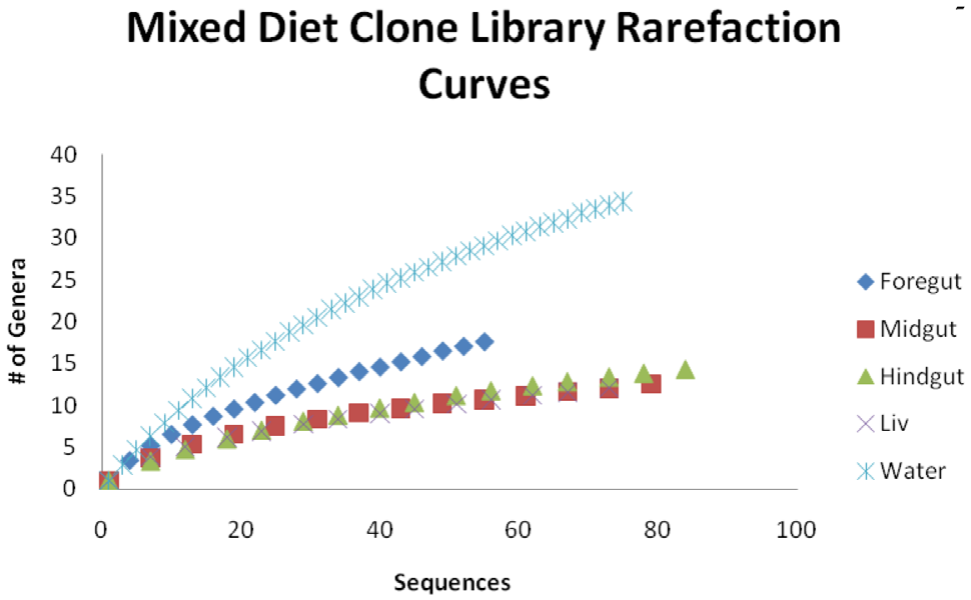
Mixed diet clone library rarefaction curves with OTUs binned to genus. Rarefaction curves were calculated [36], allowing comparison of species richness across communities with different sample sizes by plotting the species richness as a function of the number of sequences.

To better understand changes in microbial community diversity, Simpson’s diversity index and Choa_1_ estimations of diversity were applied to OTUs distributions (Table 1). Both indices revealed that tank water contained the most diverse microbial community. Diversity decreased sequentially in the foregut, midgut and hindgut, as these microbial communities were more specialized (Fig. 1 and Sup. Tables 1–5), with the lowest diversity detected in the AL and midgut. A considerable difference in OTU distribution and diversity between the tank water (65.7) and the AL (28.9) was detected using Choa_1_. These findings were further verified by the rarefaction analysis, which demonstrated that tank water had the highest detectable species richness and had not reached stationarity after ∼90 sequences, strongly suggesting that more intensive sampling would reveal a greater degree of species richness in the aquarium tank water (Fig. 2). Rarefaction curves for the AL, midgut, and hindgut were very similar, despite having very different predicted species richness based on Chao_1_ estimations (Fig. 2). This discrepancy may be a function of the weight Chao_1_ estimations places on unique (occurring once) and rare (occurring twice) OTUs in the analysis.

**Table 1:** Statistical analysis of 16S rRNA gene libraries.

	Tank Water	Foregut	Midgut	Hindgut	AL
**S_obs_**	37	18	13	15	13
**S_Chao1_**	65.7	46.9	21.3	17.6	28.9
***Coverage***	*56.48%*	*38.4%*	*61.2%*	*85.2%*	*45%*
**Simpson’s diversity** **index- D**	0.165	0.084	0.023	0.027	0.029

Simpson diversity index and Choa_1_ nonparametric estimates were used to compare species diversity in the different regions of the GI tract. For Chao_1_ phylotype richness estimations, OTUs were binned to genera.

### Distribution of Putative Diazotrophic and Cellulolytic Species

**Figure 3:**
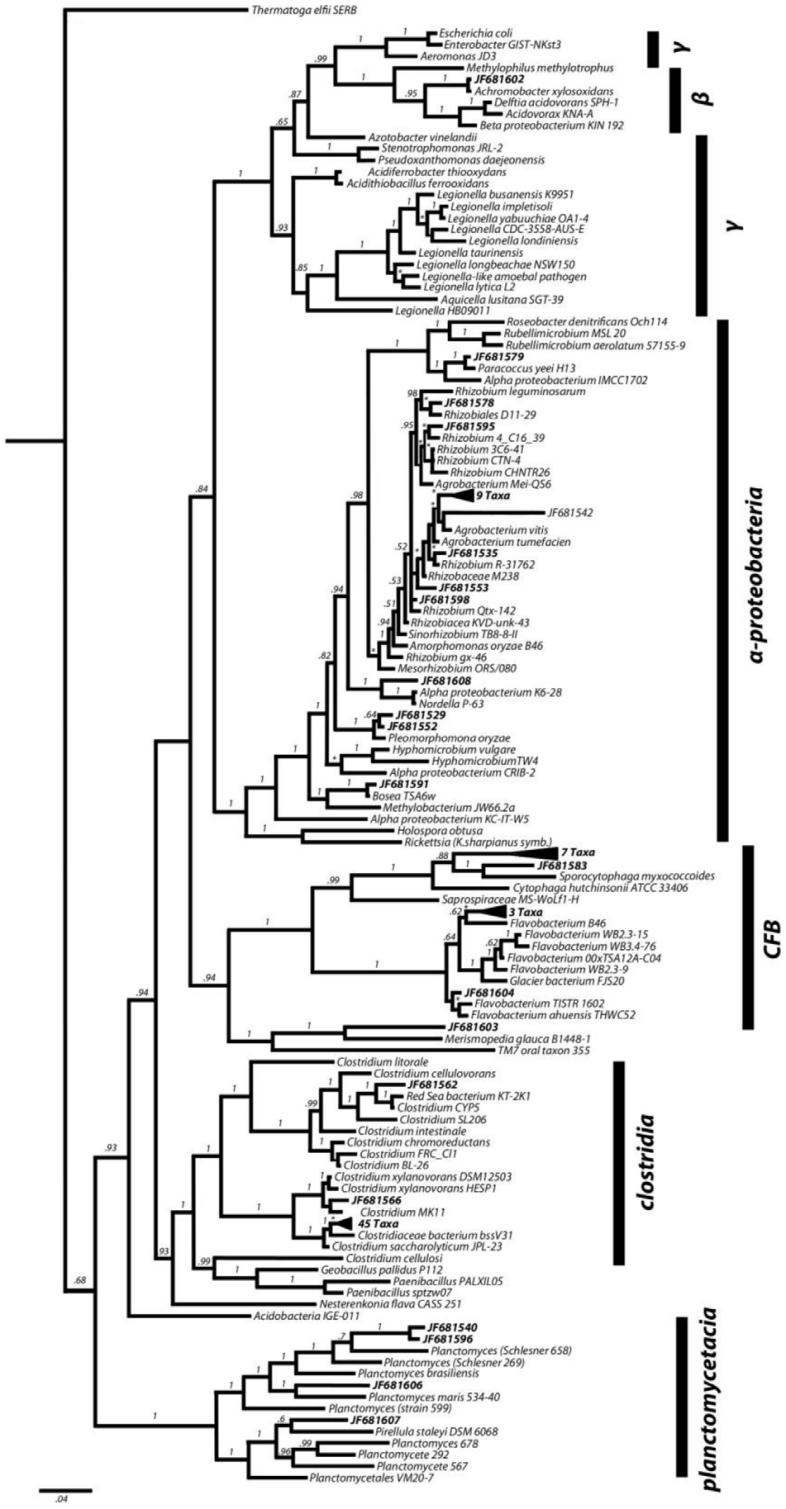
Phylogenetic tree showing the relationship of sequences identified in the midgut clone library. The tree was constructed using near full length (>1200 bp) 16S rRNA sequences using Bayesian inference (ngen = 15 000 000; BI = 12 500). Known sequences sharing highest sequence similarity were included in the phylogeny. Branch labels represent posterior probabilities (* denote branches with ≥97% posterior probability).

Distinct and diverse putative diazotrophic communities were identified in both the tank water and GI tract of *P. nigrolineatus*. The most diverse community was detected in tank water, which comprised many species of the *Beta*- and *Alpha*- subclasses of *Proteobacteria* (Sup. Table 5). The most predominant OTU shared highest sequence similarity to *Roseatales depolymerans*, but, other *Betaproteobacteria* including *Azonexus, Pelomonas, Ideonella,* and *Azospira sp.* were also detected. Sequences with the closest similarity to nitrogen-fixing bacteria of the *Alphaproteobacteria* were less abundant, but, included *Rhizobium, Agrobacterium, Sinorhizobium,* and *Devosia*. In the GI tract and AL, the putative diazotrophic communities were almost exclusively members of the *Alphaproteobacteria* (Fig. 3). Similar to the tank water, these were predominantly representatives of *Agrobacteriun, Rhizobium, Sinorhizobium,* and *Pleomorphomonas* species.

Putative cellulolytic species were identified in all libraries excluding the foregut. Diversity of potential cellulolytic microbes was low, and a number of OTUs had relatively low sequence similarity to known cellulolytic species. These included sequences closely related to *Cytophaga hutchinsonii* (89–92%) and *Clostridium xylanovorans* (95%) recovered from the midgut as well as *Sporocytophaga myxococcoides* (91–92%) recovered from both the midgut (Fig. 3) and hindgut. However, OTUs with high sequence similarity to known cellulose degraders were detected in the hindgut and AL clone libraries including *Nesterenkonia flava* (99%), *Bacteroides xylanolyticus* (99%), and *Paenibacillus* sp. PALXIL05 (98%). Only two putative cellulolytic species were identified in the tank water, *Paenibacillus* sp. PALXIL05 (the most frequently recovered OTU from the tank water, approximately ∼8% of the total clones) and *Cytophaga hutchinsonii.* Midgut and AL 16S rRNA gene libraries were dominated by a phylotype with high sequence similarity to *Clostridium saccharolyticum*, which comprised ∼52% and ∼40% of the clone libraries, respectively.

### Phylogenetic Analysis

To better visualize OTU diversity in the various tissue regions, phylogenies were reconstructed using Bayesian inference. Included in the analyses were all cloned sequences (>400) as well as retrieved sequences that shared the greatest similarity over the full 1.2 Kb. Phylogenetic analyses for all samples are available in the supplementary material and the midgut analysis is shown in Fig.3. With the exception of tank water, OTUs generally had highest similarity clustered around few bacterial classes including *Rhizobium/Agrobacterium* spp. of *Alphaproteobacteria*, *Clostridia*, and *Flavobacteria* (Fig. 3. and Supp. Fig. 1–3). Examination of terminal branch sister taxa validated the BLASTn search and OTU binning as most clones clustered near their closest matches. Most branches were well supported with posterior probabilities greater than 97%. The midgut analysis indicated that 45 OTUs with closest similarity to *Clostridium* were detected, and from the phylogenetic analysis it appears that these phylotypes were very similar to cultured representatives within the clade. However, within the *Rhizobium* clade, phylotype JF681542 (and 9 other phylotypes) appears to be highly novel as their similarity to known species is less than 97% of the 16S rRNA gene.

## Discussion

*P. nigrolineatus* imbibe wood in their natural environment and in a laboratory setting, providing a long GI tract with many microenvironments suitable for bacterial colonization enriched with cellulose and other wood components. In this study, we examined the enteric microbial community of *P. nigrolineatus* fed a mixed diet in laboratory aquaria, via 16S rRNA clone library construction. The aim of our study was to explore the microbial ecology of the *P. nigrolineatus* GI tract and identify cellulolytic and nitrogen-fixing community members that may play a role in the fish microbiome.

Microbial community diversity was highest in the tank water and decreased distally through the GI tract, with the lowest species richness observed in the hindgut and midgut according to Chao_1_ and Simpson’s diversity indices. It is unclear whether this pattern in community diversity is typical of *Osteichthyes* in general, as the development of a resident microbial community is highly complex [34]. Furthermore, most studies typically sample the entire GI tract (including the digesta) [35] or partition samples to analyze the autochthonous communities independently [36], [37], [38]. Using 16S rRNA gene analysis, Moran et al. [39] identified that microbial diversity in the marine herbivorous fish *Kyphosus sydneyanus* increased in the distal intestine. In contrast we detected a >60% reduction in predicted number of genera between the foregut (S_chao1_ = 46.9) and the hindgut (S_chao1_ = 17.6). Allocthonous communities associated with the digesta have been shown to vary little along the length of the GI tract [40], which suggests variations in overall species richness and diversity is driven by changing autochthonous communities, perhaps as a response to niche partitioning within the GI tract.

The microbial communities identified in the sampled tissues were distinct from the tank water in regard to species richness and composition. The tank water community was very diverse (S_obs_ = 37; S_chao1_ = 57.3) compared to the fish GI tract, with most OTUs belonging to the *Betaproteobacteria*. These *Betaproteobacteria* OTU types were completely absent from the GI tract analysis, qualitatively indicating the presence of a very different microbial community. Similarly, the predominant intestinal clones (*Clostridiacae, Cytophagia-Flavobacterium-Bacteroides,* and *Alphaproteobacteria*) comprised only a small proportion of the tank water clone libraries. These findings differ from previous studies that have demonstrated that the predominant OTUs were the same for the intestines and rearing water [41]. This was observed to be particularly true for fish species, like *P*. *nigrolineatus*, that lack acidic stomachs dedicated to digestion [42].

*P. nigrolineatus* examined in this study were imported without routine antibiotic treatment and conditions (temperature, light) were provided similar to those found in their natural environment. We recognize that the collection, transport, and maintenance in laboratory aquaria play a role in affecting the indigenous microbial communities present in comparison to wild animals. However, the detection of specialized microbial communities in different regions of the *Panaque* GI tract, strongly supports a view that a core microbiome selected by the intestinal habitat exists. This result is similar to a recent study in zebrafish, which showed that although differences could be detected in the microbial communities within wild vs. laboratory-reared fish, there was a shared core gut microbiota that was not affected by domestication [43].

In addition to microbial community profile differences between the tank water and GI tract, each tissue region supported a distinct community profile, indicating a specialized microbial community was resident. The most specious of the tissues, the foregut, was dominated by sequences closely related to species of *Legionella*, *Clostridia*, and *Hyphomicrobium*. Representatives of *Legionella* and *Hyphomicrobium* are ubiquitous in nature, residing in soils, as well as fresh and wastewaters. Both genera have been shown to be associated with aquarium-reared fish as either part of the normal skin microbial community, recirculating filter microbiota (*Hyphomicrobium*) [44], [45]. Both midgut and AL 16S rRNA gene libraries were dominated by OTUs with high similarity to species capable of cellulose saccharification. Most putative cellulolytic bacteria were representatives of *Clostridiacea* or the *Cytophaga-Flavobacterium-Bacteroides* group sharing greatest sequence similarity to *Clostridiacea* bacterium bssV31, *Clostridium saccharolyticum*, and *Cytophagia hutchinsonii*. *C. saccharolyticum* does not possess any endogenous cellulases, but, has been shown to grow as a co-culture with other cellulolytic species increasing the rate of cellulose degradation, as well as the growth rate of their cellulolytic counterpart [46].

The *P. nigrolineatus* GI tract contains several potential diazotrophic species, which would enhance microbial community growth on woody material by reducing atmospheric dinitrogen to assimilatable ammonia. Interestingly, the phylogenetic analysis of midgut and the other tissues (Fig. 3 and Supplementary Figures 1–3) suggested the presence of a number of novel putative nitrogen fixers and may provide new insight into the microbial ecology of this group. Our analysis shows that *P. nigrolineatus* possess an enteric putatively-diazotrophic community that is apparently unique amongst fish. Several species of *Agrobacterium/Rhizobium* and *Amorphomonas* were detected in the tank water as well as GI tract tissue, with the highest abundance in the midgut and AL. The dominant OTUs shared highest sequence similarity with free-living nitrogen-fixing *Agrobacterium tumefaciens,* and *Amorphomonas oryzae*. *Agrobacterium* has been detected in the intestinal mucosa and rearing waters of zebra fish and the summer flounder, *Paralicthys dentatus*, but, at extremely low levels (<1–3% of total clones or isolates) [47], [48]. This is the first report of diazotrophic *Alphaproteobacteria* being the dominant clones identified in the intestines of fish. In ongoing studies, we have detected both *nifH* expression and nitrogenase within the GI tract, which are consistent with the presence of an active nitrogen-fixing community (McDonald, Watts, and Schreier, in preparation).

*P. nigrolineatus* possesses a unique tissue structure, the AL, which is associated with the intestines and may play some role in digesta processing. The structure is physically attached to the intestines midway between the stomach and anus at a point where the intestines switch from clockwise to counterclockwise coiling [49]. The structure is divided into distinct lobes and is highly vascularized with many capillaries leading to the intestinal wall but, as yet, no function has been designated. The community detected in the AL of *P. nigrolineaus* shares a high degree of functional homology to those detected in several species of *Tetraponera* ants. These communities are often dominated by species of *Rhizobium, Flavobacterium*, and *Methylobacterium*, [50] all of which are represented in the AL clone library of *P. nigrolineatus*. While the role of the AL in *P. nigrolineatus* needs to be elucidated, similarities to the ant bacterial pouch warrant further examination.

Any molecular analysis of mixed microbial communities is subject to well-characterized biases. As such, little information can be derived from the absolute count of each recovered OTU. The ratios of template to products in multi-template PCR can be affected by the use of degenerate primers, starting template concentration, G+C content of the priming region, amplicon fragment length, and number of cycles [51], [52]. Similarly, this bias is exacerbated during cloning as ligation efficiencies can be affected by fragment size and G+C content. In addition, PCR induced artifacts can result in the overestimation of species richness [53]. Although these limitations can hamper quantitative analysis, assuming that the same levels of bias occur throughout the fish tissue analysis our study was able to determine and measure significant differences in the microbial community from the different GI tract regions.

Our results demonstrate the presence of a resident enteric microbial community of *P. nigrolineatus* that is unique amongst fish characterized to date and consistent with a highly enriched cellulose diet. The presence of phylotypes with high 16S rRNA gene sequence similarity to microorganisms having the capacity to carry out cellulolytic and nitrogen-fixing activities in the mid- and hindguts indicates adaptation to an enriched cellulose- and nitrogen-limiting environment. It is unlikely that these libraries represent transient communities as none of the enteric region communities are consistent with that observed in the tank water. Similarly, there is a high degree of heterogeneity between tissue regions in regards to species richness and composition. As the fish have been shown to gain no direct energy benefit from imbibing wood [6], [9], [10], it is conceivable that the resident cellulolytic and nitrogen-fixing microorganisms of the GI tract are important when utilizing a detritivorous dietary strategy by providing essential components, e.g. amino acids and vitamins, in a manner similar to host-microbe interactions in other systems [54]. This would be particularly advantageous for a species that has evolved specialized mouth musculature and teeth that are adapted for wood consumption [2], [3], [4]. Although molecular analyses have proven extremely valuable in inferring the metabolic capacities of mixed microbial communities, additional studies are ongoing to verify the role of microorganisms in both the degradation of lignocellulose and other possible positive interactions with the host.

## Supporting Information

Figure S1 **Phylogenetic tree showing the relationship of 16S rRNA sequences identified in the foregut clone library.** The tree was constructed using near full length (>1200 bp) 16S rRNA sequences Bayesian inference (ngen = 10 000 000; BI = 12 500). Known sequences sharing highest sequence similarity were included in the phylogeny. Branch labels represent posterior probabilities (* denote branches with ≥97% posterior probability).
(TIF)

Figure S2 **Phylogenetic tree showing the relationship of 16S rRNA sequences identified in the hindgut clone library.** The tree was constructed using near full length (>1200 bp) 16S rRNA sequences Bayesian inference (ngen = 10 000 000; BI = 12 500). Known sequences sharing highest sequence similarity were included in the phylogeny. Branch labels represent posterior probabilities (* denote branches with ≥97% posterior probability).
(TIF)

Figure S3 **Phylogenetic tree showing the relationship of 16S rRNA sequences identified in the AL clone library.** The tree was constructed using near full length (>1200 bp) 16S rRNA sequences Bayesian inference (ngen = 10 000 000; BI = 12 500). Known sequences sharing highest sequence similarity were included in the phylogeny. Branch labels represent posterior probabilities (* denote branches with ≥97% posterior probability).
(TIF)

Table S1 **Foregut clone library binned to closest match using NCBI BLASTn algorithm.**
(PDF)

Table S2 **Midgut clone library binned to closest match using NCBI BLASTn algorithm.**
(PDF)

Table S3 **Hindgut clone library binned to closest match using NCBI BLASTn algorithm.**
(PDF)

Table S4 **Auxiliary lobe clone library binned to closest match using NCBI BLASTn algorithm.**
(PDF)

Table S5 **Tank water clone library binned to closest match using NCBI BLASTn algorithm.**
(PDF)

## References

[1] Armbruster JW (2004) Phylogenetic relationships of the suckermouth armoured catfishes (Loricariidae) with emphasis on the *Hypostominae* and the *Ancistrinae.* . Zoological Journal of the Linnean Society 141: 1–80.

[2] Schaefer SA, Stewart DJ (1993) Systematics of *Panaque dentex* species group (Siluriformes, *Loricariidae*) wood-eating armored catfishes from tropical South America. Ichthyol Explor Freshwaters 4: 309–342.

[3] Geerinckx T, De Poorter J, Adriaens D (2007) Morphology and development of teeth and epidermal brushes in loricariid catfishes. J Morphology 268: 805–814. 10.1002/jmor.10547 17626257

[4] Lujan NK, Armbruster JW (2012) Morphological and functional diversity of the mandible in suckermouth armored catfishes (Siluriformes: *Loricariidae*). J. Morphology 273: 24–39. 10.1002/jmor.11003 21960029

[5] Nonogaki H, Nelson JA, Patterson WP (2007) Dietary histories of herbivorous loricariid catfishes: evidence from d13C values of otoliths. Environ Biol Fishes 78: 13–21.

[6] Lujan NK, German DP, Winemiller KO (2011) Do wood-grazing fishes partition their niche?: morphological and isotopic evidence for trophic segregation in Neotropical *Loricariidae*. Functional Ecology: 1327–1338.

[7] Araujo-Lima CARM, Forsberg BR, Victoria R, Martinelli L (1986) Energy sources for detritvorous fishes in the Amazon. Science 234: 1256–1258. 17778007 10.1126/science.234.4781.1256

[8] Nelson JA, Wubah DA, Whitmer ME, Johnson EA, Stewart DJ (1999) Wood-eating catfishes of the genus *Panaque*: gut microflora and cellulolytic enzyme activities. J Fish Biol 54: 1069–1082.

[9] German D (2009) Inside the guts of wood-eating catfishes: can they digest wood? J Comp Phy B 179: 1011–1023. 10.1007/s00360-009-0381-1 PMC2762535 19562350

[10] German D, Bittong R (2009) Digestive enzyme activities and gastrointestinal fermentation in wood-eating catfishes. J Comp Phys B 179: 1025–1042. 10.1007/s00360-009-0383-z PMC2762538 19568757

[11] Tsai Y-L, Olson BH (1992) Rapid method for separation of bacterial DNA from humic substances in sediments for polymerase chain reaction. Appl Environ Microbiol 58: 2292–2295. 1386212 10.1128/aem.58.7.2292-2295.1992 PMC195770

[12] Lynd LR, Weimer PJ, van Zyl WH, Pretorius IS (2002) Microbial cellulose utilization: Fundamentals and biotechnology. Microbiol Mol Biol Rev 66: 506–577. 12209002 10.1128/MMBR.66.3.506-577.2002 PMC120791

[13] Wilson DB (2011) Microbial diversity of cellulose hydrolysis. Curr Opin Microbiol 14: 259–263. 21531609 10.1016/j.mib.2011.04.004

[14] Leschine SB (1995) Cellulose degradation in anaerobic environments. Ann Rev Microbiol 8: 237–299. 10.1146/annurev.mi.49.100195.002151 8561466

[15] Bayer EA, Chanzy H, Lamed R, Shoham Y (1998) Cellulose, cellulases and cellulosomes. Curr Opin Struct Biol 8: 548–557. 9818257 10.1016/s0959-440x(98)80143-7

[16] Zaldivar J, Nielsen J, Olsson L (2001) Ethanol fuel production from lignocellulose: a challenge for metabolic engineering and process integration. Applied Microbiology and Biotechnology 56: 17–34. 11499926 10.1007/s002530100624

[17] Mattson WJ Jr (1980) Herbivory in relation to plant nitrogen content. Annual Review of Ecology and Systematics 11: 119–161.

[18] Matsumoto T (1976) The role of termites in an equatorial rain forest ecosystem of west Malaysia. I. Population density, biomass, carbon, nitrogen and calorific content and respiration rate. Oecologia 22: 153–178. 28308653 10.1007/BF00344714

[19] Luyten YA, Thompson JR, Morrill W, Polz MF, Distel DL (2006) Extensive variation in intracellular symbiont community composition among members of a single population of the wood-boring bivalve *Lyrodus pedicellatus* (Bivalvia: *Teredinidae*). Appl Environ Microbiol 72: 412–417. 16391072 10.1128/AEM.72.1.412-417.2006 PMC1352252

[20] Ohkuma M, Noda S, Kudo T (1999) Phylogenetic diversity of nitrogen fixation genes in the symbiotic microbial community in the gut of diverse termites. Appl Environ Microbiol 65: 4926–4934. 10543805 10.1128/aem.65.11.4926-4934.1999 PMC91663

[21] Berchtold M, Chatzinotas A, Schonhuber W, Brune A, Amann R, et al (1999) Differential enumeration and in situ localization of microorganisms in the hindgut of the lower termite mastotermes darwiniensis by hybridization with rRNA-targeted probes. Arch Microbiol 172: 407–416. 10591851 10.1007/s002030050778

[22] Billen J, Buschinger A (2000) Morphology and ultrastructure of a specialized bacterial pouch in the digestive tract of *Tetraponera* ants (Formicidae, Pseudomyrmecinae). Arthropod Struct Dev 29: 259–266. 18088931 10.1016/s1467-8039(00)00029-3

[23] Distel DL, DeLong EF, Waterbury JB (1991) Phylogenetic characterization and in situ localization of the bacterial symbiont of shipworms (Teredinidae: *Bivalvia*) by using 16S rRNA sequence analysis and oligodeoxynucleotide probe hybridization. Appl Environ Microbiol 57: 2376–2382. 1722662 10.1128/aem.57.8.2376-2382.1991 PMC183578

[24] Brune A, Friedrich M (2000) Microecology of the termite gut: structure and function on a microscale. Curr Opin Microbiol 3: 263–269. 10851155 10.1016/s1369-5274(00)00087-4

[25] Lane DJ (1991) 16S/23S rRNA sequencing. In: Stackebrandt E.G, M., editor. Nucleic acid techniques in bacterial systematics. Cambridge, United Kingdom.: John Wiley and Sons Ltd. 115–175.

[26] Ferris MJ, Muyzer G, Ward DM (1996) Denaturing gradient gel electrophoresis profiles of 16S rRNA-defined populations inhabiting a hot spring microbial mat community. Appl Environ Microbiol 62: 340–346. 8593039 10.1128/aem.62.2.340-346.1996 PMC167804

[27] DeSantis TZ, Hugenholtz P, Larsen N, Rojas M, Brodie EL, et al (2006) Greengenes, a chimera-checked 16S rRNA gene database and workbench compatible with ARB. Appl Environ Microbiol 72: 5069–5072. 16820507 10.1128/AEM.03006-05 PMC1489311

[28] Altschul SF, Gish W, Miller E, Meyers EW, Lipman DJ (1990) Basic local alignment search tool. J Mol Biol 215: 403–410. 2231712 10.1016/S0022-2836(05)80360-2

[29] Huelsenbeck JP, Ronquist F (2001) MRBAYES: Bayesian inference of phylogeny. Bioinformatics 17: 754–755. 11524383 10.1093/bioinformatics/17.8.754

[30] Simpson EH (1949) Measurements of diversity. Nature 163: 688.

[31] Chao A (1984) Non-parametric estimation of the number of classes in a population. Scand J Stat 11: 265–270.

[32] Hughes JB, Hellmann JJ, Ricketts TH, Bohannan BJM (2001) Counting the Uncountable: Statistical Approaches to Estimating Microbial Diversity. Appl Environ Microbiol 67: 4399–4406. 11571135 10.1128/AEM.67.10.4399-4406.2001 PMC93182

[33] Schloss PD, Westcott SL, Ryabin T, Hall JR, Hartmann M, et al (2009) Introducing mothur: Open-source, platform-independent, community-supported software for describing and comparing microbial communities. Appl Environ Microbiol 75: 7537–7541. 19801464 10.1128/AEM.01541-09 PMC2786419

[34] Ringo E, Birkbeck TH (1999) Intestinal microflora of fish larvae and fry. Aquaculture Research 30: 73–93.

[35] Martin-Antonio B, Manchado M, Infante C, Zerolo R, Labella A, et al (2007) Intestinal microbiota variation in Senegalese sole (*Solea senegalensis)* under different feeding regimes. Aquaculture Research 38: 1213–1222.

[36] Dhanasiri AK, Brunvold L, Brinchmann MF, Korsnes K, Bergh O, et al (2011) Changes in the intestinal microbiota of wild Atlantic cod *Gadus morhua L.* upon captive rearing. Microb Ecol 61: 20–30. 20424834 10.1007/s00248-010-9673-y

[37] Kim DH, Brunt J, Austin B (2007) Microbial diversity of intestinal contents and mucus in rainbow trout (*Oncorhynchus mykiss*). J Appl Microbiol 102: 1654–1664. 17578431 10.1111/j.1365-2672.2006.03185.x

[38] Wu S, Gao T, Zheng Y, Wang W, Cheng Y, et al (2010) Microbial diversity of intestinal contents and mucus in yellow catfish (*Pelteobagrus fulvidraco)* . Aquaculture 303: 1–7.

[39] Moran D, Turner SJ, Clements KD (2005) Ontogenetic development of the gastrointestinal microbiota in the marine herbivorous fish *Kyphosus sydneyanus* . Microbial Ecology 49: 590–597. 16041474 10.1007/s00248-004-0097-4

[40] Merrifield DL, Burnard D, Bradley G, Davies SJ, Baker RTM (2009) Microbial community diversity associated with the intestinal mucosa of farmed rainbow trout (*Oncoryhnchus mykiss Walbaum)* . Aquaculture Research 40: 1064–1072.

[41] Romero J, Navarrete P (2006) 16S rDNA-based analysis of dominant bacterial populations associated with early life stages of coho salmon (*Oncorhynchus kisutch*). Microb Ecol 51: 422–430. 16598631 10.1007/s00248-006-9037-9

[42] Sera H, Ishida Y (1972) Bacterial flora in the digestive tracts of marine fish-III. Classification of isolated bacteria. Bulletin of the Japanese Society of Scientific Fisheries 38: 853–858.

[43] Roeselers G, Mittge EK, Stephens WZ, Parichy DM, Cavanaugh CM, et al (2011) Evidence for a core gut microbiota in the zebrafish. ISME J 5: 1595–1608. 21472014 10.1038/ismej.2011.38 PMC3176511

[44] Mudarris M, Austin B (1988) Quantitative and qualitative studies of the bacterial microflora of turbot, *Scophthalmus maximus L.,* gills. J Fish Biol 32: 223–229.

[45] Sugita H, Nakamura H, Shimada T (2005) Microbial communities associated with filter materials in recirculating aquaculture systems of freshwater fish. Aquaculture 243: 403–409.

[46] Murray WD (1986) Symbiotic relationship of *Bacteroides cellulosolvens* and *Clostridium saccharolyticum* in cellulose fermentation. Appl Environ Microbiol 51: 710–714. 16347034 10.1128/aem.51.4.710-714.1986 PMC238952

[47] Rawls JF, Samuel BS, Gordon JI (2004) Gnotobiotic zebrafish reveal evolutionarily conserved responses to the gut microbiota. Proc Natl Acad Sci U S A 101: 4596–4601. 15070763 10.1073/pnas.0400706101 PMC384792

[48] Eddy SD, Jones SH (2002) Microbiology of summer flounder *Paralichthys dentatus* fingerling production at a marine fish hatchery. Aquaculture 211: 9–28.

[49] Dehn A (2010) Physiological, histological, and morphological evidence of microbial symbiosis in a xylophagous catfish *Panaque nigrolineatus* Towson: Towson University.

[50] Russell JA, Moreau CS, Goldman-Huertas B, Fujiwara M, Lohman DJ, et al (2009) Bacterial gut symbionts are tightly linked with the evolution of herbivory in ants. Proc Natl Acad Sci U S A 106: 21236–21241. 19948964 10.1073/pnas.0907926106 PMC2785723

[51] Polz MF, Cavanaugh CM (1998) Bias in template-to-product ratios in multitemplate PCR. Appl Environ Microbiol 64: 3724–3730. 9758791 10.1128/aem.64.10.3724-3730.1998 PMC106531

[52] Suzuki MT, Giovannoni SJ (1996) Bias caused by template annealing in the amplification of mixtures of 16S rRNA genes by PCR. Appl Environ Microbiol 62: 625–630. 8593063 10.1128/aem.62.2.625-630.1996 PMC167828

[53] Acinas SG, Sarma-Rupavtarm R, Klepac-Ceraj V, Polz MF (2005) PCR-induced sequence artifacts and bias: insights from comparison of two 16S rRNA clone libraries constructed from the same sample. Appl Environ Microbiol 71: 8966–8969. 16332901 10.1128/AEM.71.12.8966-8969.2005 PMC1317340

[54] Bäckhed F, Ley RE, Sonnenburg JL, Peterson DA, Gordon JI (2005) Host-bacterial mutualism in the human intestine. Science 307: 1915–1920. 15790844 10.1126/science.1104816

