# Supplementary material for: Phylogenetic Analysis of Microbial Communities in Different Regions of the Gastrointestinal Tract in Panaque nigrolineatus, a Wood-Eating Fish
Source: PLoS One. 2012 Oct 25;7(10):e48018. doi: 10.1371/journal.pone.0048018 (PMC3485024; doi:10.1371/journal.pone.0048018)
Supplement: Table S1 — Foregut clone library binned to closest match using NCBI BLASTn algorithm. (PDF) [file pone.0048018.s004.pdf]

**Bacterial Phylum/Class**	**Related Species**	**Accession #**	**Similarity (%)**	**Clones**
**Alphaproteobacteria**	*Agrobacterium* sp. Mei-Qs6	EF090606	99	1
	Hyphomicrobium vulgare	AB543807	95-98	7
	Hyphomicrobium sp. TW4	AY934492	96	3
	Nordella sp. P-63	AM411927	96	3
	Rubellimicrobium aerolatum	EU338486	95	1
	Rhizobiaceae bacterium KVD-unk-43	DQ490336	95-96	2
	Rhizobium sp. 3-4	FJ598330	98	1
	Pleomorphomonas oryzae	AB159685	99	1
	Agrobacterium tumefaciens	DQ993282	96	1
	Mesorhizobium sp. ORS1080	AJ295082	91	1
	Holospora obtusa	X58198	92	1
	Alpha proteobacterium CRIB-02	DQ123619	98	2
**Gammaproteobacteria**	*Acidithiobacillus ferrooxidans*	AJ459800	85	1
	Legionella busanensis	NR_025196	91	6
	Legionella taurinensis	DQ667196	84-86	2
	Legionella longbeachae	FN650140	91	1
	Legionella impletisoli	AB233209	87	1
	Legionella sp. HB09011	GU319886	85	1
	Legionella yabuuchiae	AB233212	91	1
	Legionella lytica	NR_026334	95	1
**Clostridia**	*Clostridium* sp. SL206	EU816420	94	1
	Clostridium intestinale	AY781385	99	4
	Clostridium chromoreductans	AY228334	99	1
	Clostridium sp. MK11	AB275140	97	2
	Clostridium sp. CYP5	DQ479415	98	2
	Clostridium saccharolyticum	FJ957875	97	2
	Clostridium sp. FRC_Cl1	EU331374	99	1
	Clostridium sp. BL-26	DQ196630	99	1
**Flavobacteria**	*Flavobacterium* sp. TISTR	AB465580	95-98	4
**Planctomycetacia**	*Planctomyces* sp. (strain 599)	AJ231189	90-95	3
	Pirellula sp. (Schlesner 678)	X81947	85-86	2
	Planctomyces sp. (Schlesner 269)	X81953	89	1
	Planctomycete str. 292	AJ231182	91	1
**Bacilli**	*Geobacillus pallidus*	FJ808721	99	1
**TM7**	TM7 phylum sp. oral taxon 355 clone	GU410617	87	2
**Total**				67
