# Supplementary material for: Phylogenetic Analysis of Microbial Communities in Different Regions of the Gastrointestinal Tract in Panaque nigrolineatus, a Wood-Eating Fish
Source: PLoS One. 2012 Oct 25;7(10):e48018. doi: 10.1371/journal.pone.0048018 (PMC3485024; doi:10.1371/journal.pone.0048018)
Supplement: Table S2 — Midgut clone library binned to closest match using NCBI BLASTn algorithm. (PDF) [file pone.0048018.s005.pdf]

Bacterial Phylum/Class	Related Species	Accession #	Similarity (%)	Clones
**Alphaproteobacteria**	*Agrobacterium tumefaciens*	GU479944	96-98	8
	Rhizobium sp. CHNTR26	DQ337571	97	1
	Rhizobium sp. Qtx-14-2	GU201841	93	1
	Rhizobium sp. R-31762	AM403584	99	1
	Agrobacterium vitis	EF590316	90	1
	Rhizobium sp. CTN-4	FJ539087	98	1
	Rhizobilales bacterium D11-29	AM403229	99	1
	Paracoccus yeeii strain H13	AY014178	99	1
	Sporocytophaga myxococcoides	NR_025463	91	1
	Bosea sp. TSA6w	AB542375	99	1
	Rhizobium sp. 4_C16_39	EF540509	98	1
	Amorphomonas oryzae	EU770254	98	1
	Nordella sp. P-63	AM411927	93	1
**Betaproteobacteria**	*Achromobacter xylosoxidans*	AF531768	99	1
**Clostridia**	*Clostridium saccharolyticum*	FJ957875	97-98	45
	Clostridium sp. CYP5	DQ479415	95	1
	Clostridium xylanovorans	NR_028740	95	1
**Flavobacteria**	*Flavobacterium* sp. WB2.3-15	AM934646	97	2
	Flavobacterium sp. WB3.4-76	AM934664	95	1
	Flavobacterium sp. TISTR 1602	AB465580	98	1
**Planctomycetacia**	*Planctomyces* sp. (Schlesner 269)	X81953	90	2
	Planctomyces maris	NR025327	90	1
	Planctomycetales bacterium VM20-7	AB558583	88	1
**Bacteroidetes**	*Cytophaga hutchinsonii*	CP000383	89-92	8
	Pleomorphomonas oryzae	AY928261	98	2
**Total**				86
