# Supplementary material for: Phylogenetic Analysis of Microbial Communities in Different Regions of the Gastrointestinal Tract in Panaque nigrolineatus, a Wood-Eating Fish
Source: PLoS One. 2012 Oct 25;7(10):e48018. doi: 10.1371/journal.pone.0048018 (PMC3485024; doi:10.1371/journal.pone.0048018)
Supplement: Table S3 — Hindgut clone library binned to closest match using NCBI BLASTn algorithm. (PDF) [file pone.0048018.s006.pdf]

Bacterial Phylum/Class	Related Species	Accession #	Similarity (%)	Clones
**Alphaproteobacterium**	*Rhizobiaceae* bacterium KVD-unk-43	DQ490336	96	3
	Agrobacterium tumefaciens	GU479944	96-98	2
	Rhizobium sp. gx-46	FJ823050	96	2
	Alpha proteobacterium KC-IT-W5	FJ711211	98	1
	Sinorhizobium sp. TB8-8-II	AY599696	95	1
**Betaproteobacteria**	Beta proteobacterium KIN192 *(Comanodacea)*	AY136099	98-99	13
	Acidovorax sp. KNA-A	AB539974	99	2
**Gammaproteobacteria**	*Aeromonas* sp. JD3	GU566315	99	2
	Pseudoxanthomonas daejeonensis	AY550264	98-99	3
	Legionella londiniensis	AB514842	92	1
**Flavobacteria**	*Flavobacterium* sp. B4	EU194891	95-97	39
	Flavobacterium sp. TISTR 1602	AB465580	95-98	10
	Flavobacterium ahuensis	GQ284450	97	4
	Flavobacterium sp. 001xTSA12A_C04	HM113609	96	1
	Flavobacterium sp. WB2.3-9	AM934644	97	1
	Glacier bacterium FJS20	AY315160	97	1
**Planctomycetacia**	*Pirellula staleyi*	CP001848	90	1
	Planctomyces maris	NR025327	88	1
	Planctomyces sp.(Schlesner 658)	X81954	96	1
**Bacteroidetes**	*Sporocytophaga myxococcoides*	NR025463	92	1
**Bacilli**	*Paenibacillus* sp. Sptzw07	GU377098	98	1
	Paenibacillus sp. PALXIL05	DQ407279	98	1
**Sphingobacteria**	*Saprospiraceae* bacterium MS-Wolf1-H	AJ786322	85	1
**Acidobacteria**	*Acidobacteria* bacterium IGE-011	GU187027	88	1
**Total**				94
