# Supplementary material for: Phylogenetic Analysis of Microbial Communities in Different Regions of the Gastrointestinal Tract in Panaque nigrolineatus, a Wood-Eating Fish
Source: PLoS One. 2012 Oct 25;7(10):e48018. doi: 10.1371/journal.pone.0048018 (PMC3485024; doi:10.1371/journal.pone.0048018)
Supplement: Table S4 — Auxiliary lobe clone library binned to closest match using NCBI BLASTn algorithm. (PDF) [file pone.0048018.s007.pdf]

**Bacterial Phylum/Class**	**Related Species**	**Accession #**	**Similarity (%)**	**Clones**
**Alphaproteobacterium**	*Agrobacterium tumefaciens*	DQ993282	96-97	12
	Amorphomonas oryzae	AB233493	97-98	8
	Rhizobium sp. CTN-4	FJ539087	98	5
	Rape rhizosphere bacterium tsb077	AJ295454	99	4
	Rhizobiaceae bacterium M238	AB461714	98	1
	Agrobacterium sp. AL060301_26	FJ593844	97	1
	Catellibacterium A1-9	EU313813	97	1
	Methylobacterium zatmanii	AB175647	99	1
**Gammaproteobacteria**	*Stenotrophomonas maltophilia*	GU254017	99	1
	Legionella sp. CDC-3558-AUS-E	FJ236837	87	1
	Enterobacter sp. GIST-NKst3	EF489445	99	1
**Flavobacteria**	*Flavobacterium* sp. B46	EU194891	96	3
	Flavobacterium ahuensis	GQ284450	97	3
**Clostridia**	*Clostridium saccharolyticum*	FJ957875	97-98	33
	Clostridium sp. BL-26	DQ196630	99	1
	Clostridium tunisiense	AY187622	95	1
**Planctomycetacia**	*Planctomyces* sp. (strain 599)	AJ231189	91	1
**Bacteroidetes**	*Bacteroides xylanolyticus*	DQ497992	99	2
**Actinobacteria**	*Nesterenkonia flava*	EF680886	99	2
**Cyanobacteria**	*Merismopedia glauca*	X94705	84	1
**Total**				83
