# Supplementary material for: Phylogenetic Analysis of Microbial Communities in Different Regions of the Gastrointestinal Tract in Panaque nigrolineatus, a Wood-Eating Fish
Source: PLoS One. 2012 Oct 25;7(10):e48018. doi: 10.1371/journal.pone.0048018 (PMC3485024; doi:10.1371/journal.pone.0048018)
Supplement: Table S5 — Tank water clone library binned to closest match using NCBI BLASTn algorithm. (PDF) [file pone.0048018.s008.pdf]

Bacterial Phylum/Class	Related Species	Accession #	Similarity (%)	Clones
**Alphaproteobacteria**	*Agrobacterium tumefaciens*	GU479944	96-99	3
	Sphingomonadaceae bacterium PB307	AB220143	88	1
	Rhodobacter blasticus	DQ342322	97	1
	Devosia sp. T15	FJ687971	98	1
	Bacterium 081631 *(Sphingomonas)*	AB202227	90	1
	Alpha proteobacterium BAC233	EU180520	95	1
	Sinorhizobium sp. XLL-7	EF027003	95	1
	Agrobacterium tumefaciens	GU479944	97	1
	Sphingomonas sp. SIA181-1A1	AF395032	89	1
**Betaproteobacterium**	*Roseateles depolymerans*	AM990542	94-99	6
	Beta proteobacterium A0618	AF236006	99	1
	Beta proteobacterium KIN192	AY136099	95-98	2
	Beta proteobacterium OcN1	AF331976	96	2
	Methylophilus leisingeri	AB193725	97	2
	Acidovorax sp. R-25052	AM084039	97	1
	Aquabacterium sp. P-136	AM412127	99	2
	Vogesella sp. TPS6	FJ821602	89	1
	Azonexus fungiphilus	NR_024853	96-99	3
	Thiobacter subterraneus	NR_024834	92	1
	Beta proteobacterium OS-19	AB076848	98	1
	Burkholderiales bacterium MSMB34	EF114435	96	1
	Chitinimonas taiwanensis	GQ284441	99	1
	Pelomonas sp. On15	DQ291149	98	1
	Pelomonas puraquae	AM501441	95	1
	Dechloromonas sp. PC1	AY126452	97	2
	Pelomonas aquatic	AM501435	98	1
	Aquabacterium hongkongensis	DQ489306	99	1
	Ideonella sp. B513	AB049107	97	1
	Ralstonia eutropha	AF027407	95	1
	Pelomonas saccharophila	FJ513082	99	2
	Azospira restricta	DQ974114	90	1
	Mitsuaria chitosanitabida	AM501442	91-94	2
	Burkholderiales bacterium TP320	EF636151	98	1
**Gammaproteobacteria**	*Pseudomonas* sp. JPB-1.12	EU652471	96	1
	Pseudomonas sp. BBCT8	DQ337559	99	1
**Deltaproteobacteria**	*Chondromyces crocatus*	FJ176772	90	1
**Flavobacteria**	*Flavobacterium* sp. TISTR 1602	AB465580	95-97	4
	Flavobacterium sp. ARSA-108	GU295968	96	1
	Flavobacterium sp. B46	EU194891	94	1
	Flavobacteriaceae bacterium R2A-16	EU581834	93	1
**Clostridia**	*Clostridium* sp. PPf35E10	AY548785	94	1
	Sporacetigenium mesophilum	AY682207	96	1
	Red Sea bacterium KT-2K1	AJ309522	98	1
	Clostridium sp. Kas202-1	AB114252	96	1
	Tepidibacter formicigenes	FN666239	94-95	2
**Planctomycetacia**	*Planctomycete* A-2	AM056027	78-82	2
	Gemmata-like str. CJuql4	AF239693	99	1
**Bacteroidetes**	*Bacteroidetes* bacterium N2	AB540001	92-93	2
	Cytophaga hutchinsonii	CP000383	90	1
	Bacteroidetes bacterium 10AO	GU117703	88	1
	Bacterium TG141	AB308367	93	2

	Bacteroidetes bacterium ArSB	AB539997	96	1
**Bacilli**	*Paenibacillus* sp. PALXIL05	DQ407279	95-98	7
	Paenibacillus sp. Sptzw08	GU377099	98	1
**Negativicutes**	*Anaerospora hongkongensis*	AY372052	99	1
**Verrucomicrobia**	*Opitutus* sp. VeSm13	X99392	95	2
**Cyanobacteria**	*Nostoc* sp. NTK29	DQ513319	96	1
**Total**				87
